# Institution of an emergency department “swarming” care model and sepsis door-to-antibiotic time: A quasi-experimental retrospective analysis

**DOI:** 10.1371/journal.pone.0232794

**Published:** 2020-05-05

**Authors:** Ithan D. Peltan, Joseph R. Bledsoe, David Brems, Sierra McLean, Emily Murnin, Samuel M. Brown

**Affiliations:** 1 Division of Pulmonary and Critical Care Medicine, Department of Medicine, Intermountain Medical Center, Murray, UT, United States of America; 2 Division of Pulmonary and Critical Care Medicine, Department of Medicine, University of Utah School of Medicine, Salt Lake City, UT, United States of America; 3 Department of Emergency Medicine, Intermountain Medical Center, Murray, UT, United States of America; 4 Department of Emergency Medicine, Stanford Medicine, Palo Alto, CA, United States of America; 5 Department of Emergency Medicine, LDS Hospital, Salt Lake City, UT, United States of America; 6 University of Utah School of Medicine, Salt Lake City, UT, United States of America; University of Maryland School of Medicine, UNITED STATES

## Abstract

**Background:**

Prompt sepsis treatment is associated with improved outcomes but requires a complex series of actions by multiple clinicians. We investigated whether simply reorganizing emergency department (ED) care to expedite patients’ initial evaluation was associated with shorter sepsis door-to-antibiotic times.

**Methods:**

Patients eligible for this retrospective study received IV antibiotics and demonstrated acute organ failure after presenting to one of three EDs in Utah. On May 1, 2016, the intervention ED instituted “swarming” as the default model for initial evaluation of all mid- and low-acuity patients. Swarming involved simultaneous patient evaluation by the ED physician, nurse, and technician followed by a team discussion of the initial care plan. Care was unchanged at the two control EDs. A 30-day wash-in period separated the baseline (May 16, 2015 to April 15, 2016) and post-intervention (May 16, 2016 to November 15, 2016) analysis periods. We conducted a quasi-experimental analysis comparing door-to-antibiotic time for sepsis patients at the intervention ED after versus before care reorganization, applying difference-in-differences methods to control for trends in door-to-antibiotic time unrelated to the studied intervention and multivariable regression to adjust for patient characteristics.

**Results:**

The analysis included 3,230 ED sepsis patients, including 1,406 from the intervention ED. Adjusted analyses using difference-in-differences methods to control for temporal trends unrelated to the studied intervention revealed no significant change in door-to-antibiotic time after care reorganization (-7 minutes, 95% CI -20 to 6 minutes, p = 0.29). Multivariable pre/post analyses using data only from the intervention ED overestimated the magnitude and statistical significance of outcome changes associated with ED care reorganization.

**Conclusions:**

Implementation of an ED care model involving parallel multidisciplinary assessment and early team discussion of the care plan was not associated with improvements in mid- and low-acuity sepsis patients’ door-to-antibiotic time after accounting for changes in the outcome unrelated to the studied intervention.

## Introduction

Severe infection causing organ failure—sepsis—is a common problem among emergency department (ED) patients associated with substantial costs, morbidity, and mortality [1,2]. Prompt antibiotics are associated with improved sepsis outcomes [3–5], and are key features of international guidelines and regulatory mandates [6,7].

Numerous non-clinical factors influence the timing of sepsis care [8–12]. Reported approaches to overcome these barriers and improve ED sepsis care vary with respect to the type, number, and intensity of interventions. Team-based strategies meant to encourage parallel (rather than sequential) task performance and help clinicians develop a shared mental model for patients’ care are common components of these interventions. More complex and intensive interventions, however, may entail substantial costs [13] and increase the likelihood of harmful unintended effects.

We sought to investigate whether lower-intensity interventions can meaningfully improve sepsis care delivery. To accomplish this goal, we performed a retrospective, quasi-experimental analysis of sepsis door-to-antibiotic times based on a natural experiment in which ED care for all mid- and low acuity ED patients was reorganized around early, routine multidisciplinary evaluation and care planning—an approach to care sometimes termed “swarming” [14,15]— for all ED patients.

## Methods

### Study design and setting

We conducted a retrospective cohort study of septic adult patients presenting to three community EDs belonging to one health system in the Salt Lake City, Utah metropolitan area. The study EDs’ annual census ranged from approximately 21,000–25,000 patient visits. Data were collected before and after reorganization of initial patient evaluation at the intervention ED on May 1, 2016. The study employed a quasi-experimental design using data from two control EDs to control for changes in outcomes at the intervention ED unrelated to the studied care reorganization. All three EDs employed a common standardized sepsis protocol both before and after care reorganization [16].

This study was approved with waiver of informed consent by the Intermountain Healthcare Institutional Review Board. This retrospective analysis was registered on ClinicalTrials.gov (NCT03226366) prior to data abstraction or analysis.

### Study population

Adult patients (age ≥18 years) were eligible for inclusion in this study if they met sepsis criteria before discharge from a study ED and presented to a study ED between May 16, 2015 and April 15, 2016 (pre-intervention period) or May 16, 2016 and November 15, 2016 (post-intervention period). The pre- and post-intervention analysis periods were separated by a 30-day wash-in period surrounding the intervention’s launch date on May 1, 2016. Sepsis was defined by the combination of administration of IV antimicrobial while the patient was in the ED and a Sequential [Sepsis-Associated] Organ Failure Assessment (SOFA) score ≥2 points above baseline based on data available in the ED [17,18]. IV-equivalent antimicrobials–oral administration of oseltamivir, fidaxomicin, or vancomycin —were also considered eligible. Consistent with clinical practice and prior studies [19], a normal SOFA component score of 0 was assumed when data for its calculation was missing. Data available from 3 years to 24 hours prior to ED arrival was used to calculate baseline SOFA. Patients assigned the highest triage acuity score based on the five-point Canadian Triage Acuity Score (CTAS) system [20] were expected to receive an immediate multidisciplinary evaluation independent of the intervention under study and were excluded from the present study.

### Intervention

During the pre-intervention/baseline period, patients assigned a triage acuity score of 2–5 during initial triage thereafter underwent sequential assessment by a critical care technician, nurse, and physician or advanced practice clinician. Tasks performed included helping the patient change into a patient gown and settle into the ED bed (technician and/or nurse), gathering vital signs and connection to telemetry monitor (technician and/or nurse), history taking (physician and nurse), physical examination (physician and nurse), bedside clinical documentation (nurse), IV placement (technician), and phlebotomy (technician). Care delivery in the two control EDs remained unchanged during the post-intervention period.

As part of an effort to improve the efficiency of care, beginning May 1, 2016, the intervention ED reorganized initial evaluation of all patients using a simplified version of the “swarming” approach (Table 1) already applied to patients with serious trauma, ST-elevation myocardial infarction, cardiopulmonary instability, and other patients assigned the highest triage acuity score [14,15]. Under this paradigm, ED nurses signaled readiness for “swarming” of a patient by setting to green, yellow, or red an indicator light (Luxafor, Riga, Latvia) attached to their computer screen in the nursing work station. ED physicians activated the swarm by asking a “green status” nurse and a critical care technician to join them for a patient swarm. This core ED clinical team then proceeded to perform simultaneously their initial patient evaluation and clinical tasks as described above. In most circumstances, the swarm occurred concurrent with patients being “settled” into the ED room by the nurse and critical care technician but, when a physician was unavailable at the time of a patient’s initial room placement, “settling in” tasks and the remainder of swarming could occur sequentially. The physician and nurse jointly gathered a history, thereby avoiding repeating each other’s questions and allowing each clinician to learn from and build upon history obtained by the other. Initial diagnostic testing and clinical care (e.g. collection of specific laboratory tests, initiation of intravenous fluids) was begun immediately based on verbal communication of patient care orders entered concurrently in the patient’s chart. The team then discussed the initial clinical impression and plan in the patient’s presence prior to leaving the room, helping patients understand their care plan and facilitating a shared mental model amongst the clinical team regarding the diagnostic and treatment plan and the priority of each of its components. Patients were also given an opportunity to clarify their history and ask questions. Care delivery processes occurring subsequent to the initial assessment were unchanged.

**Table 1:** Potential mechanisms of effect of “swarming” on sepsis door-to-antibiotic time.

Swarming intervention component	Potential mechanisms for door-to-antibiotic time effect
Parallel patient evaluation	Decreased net time for initial clinical evaluation by all members of the clinical team
	More accurate understanding of sepsis risk for all team members
	Earlier review of initial vital signs by nurse and physician
Verbal communication of diagnostic evaluation plan	Earlier availability of diagnostic data to facilitate antibiotic initiation decision and antibiotic selection
Collection of initial specimens for laboratory testing	Earlier availability of diagnostic data to facilitate antibiotic initiation decision and antibiotic selection
Verbal communication of initial treatment plan	Earlier order for antibiotics
	Nurse immediately aware of treatment orders/plan
Creation of shared mental model for diagnostic/treatment plan	Improved prioritization of steps required to identify infection source and diagnose sepsis
	Shared sense of urgency
Patient present for discussion of diagnostic/treatment plan	Patient able confirm allergies and clarify or provide additional sepsis-relevant history

While swarming was adopted as the default care model, it was not formally mandated for all patients; we were not able to identify which specific post-implementation patients actually received their care under this paradigm. From mid-May through late September 2016, ED nurses were asked to semi-formally record each swarming event by affixing an indicator sticker to a swarming documentation worksheet. Swarming adherence was calculated daily as the number of swarming events divided by the intervention ED’s patient census. Because patient-level data were not stored and some swarming events were not recorded, swarming adherence was measured on the entire ED population rather than the specific population targeted for the present analysis and may underestimate actual adherence.

### Data collection and definitions

Patient clinical and demographic data was obtained from the Intermountain Healthcare Electronic Data Warehouse [21]. Antimicrobial administration time was abstracted from structured nursing documentation in the electronic medical record. Missing data and outlier values for ED care event times (including antibiotic administration, ED arrival, and ED departure), vital signs, mode of ED arrival, and race/ethnicity were verified by manual chart review by trained data abstractors. Among 100 randomly-selected subjects, manual chart review was also used to determine whether infection was present in the ED based on all available data. Due to very few patients having triage acuity score of 5 (the lowest value), we combined the two lowest CTAS categories (“semi-urgent” and “non-urgent”) into a single group for analysis. The first-reported ED vital signs were collected. Glasgow Coma Scale (GCS) score was dichotomized into normal (GCS 15) and abnormal (GCS ≤14). ED mode of arrival was dichotomized as medical (ambulance) or non-medical (e.g., personal vehicle or “walk in”). The Charlson Comorbidity Index and Quick SOFA (qSOFA) score were calculated as previously described [19,22,23]. Initial physician evaluation time was obtained from the ED patient tracking system as the time a physician was assigned to the patient.

### Analysis

The primary exposure was ED admission after May 1, 2016 at the intervention ED. The primary outcome was door-to-antibiotic time, defined as the time from ED registration to administration of the first eligible IV- or IV-equivalent antibiotic. Secondary outcomes were antibiotic administration within 3 hours of ED arrival, ED length of stay, time from ED registration to initial physician evaluation, and inpatient mortality. Continuous data are reported as median values with interquartile range and compared using Mann-Whitney rank-sum or Kruskal-Wallis tests as appropriate. Categorical data were compared using Chi-square tests.

**Fig 1:**
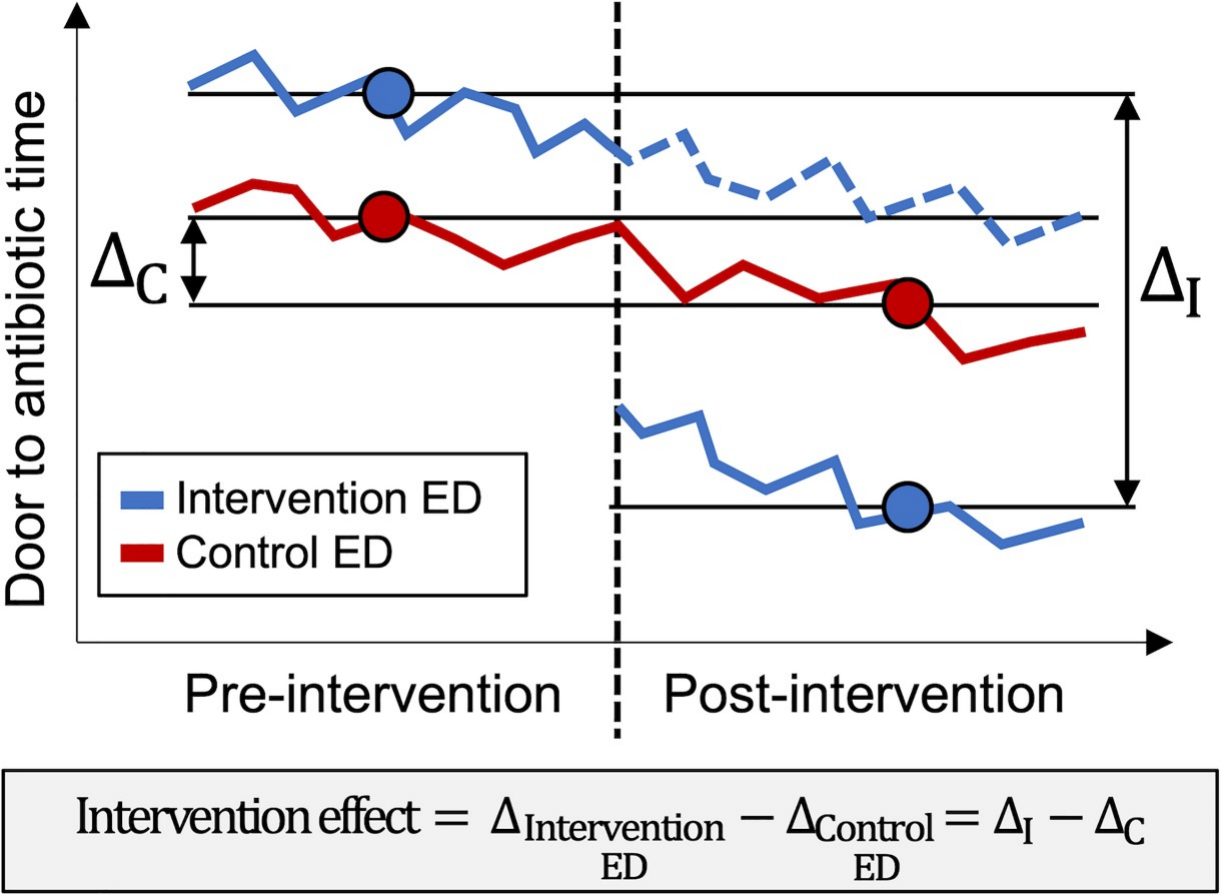
Schematic diagram of difference-in-differences analysis. Solid lines represent hypothetical observed data for each emergency department (ED); dotted lines represent the data expected without intervention. The point estimates of analysis period means are indicated by solid circles.

The primary, prespecified analysis employed a well-established quasi-experimental method called difference-in-differences analysis. Specifically, we used multivariable linear or logistic regression with robust standard error estimation to compare post-intervention and pre-intervention outcomes adjusted for a prespecified set of confounders potentially associated with time of year, study ED presentation, and door-to-antibiotic time: age, sex, Charlson Comorbidity Index, initial GCS ≤14, initial systolic blood pressure, ED triage acuity score, and arrival to the ED via ambulance. Inclusion in the multivariable model of dichotomous indicator variables for ED arrival period (pre-intervention versus post-intervention), study site (intervention versus control), and an interaction term between arrival period and site provides an estimate of the effect of the intervention with the studied outcomes that excludes temporal changes—“secular trends”— in the measured outcome unrelated to the study intervention (Fig 1). To assess the impact of not accounting for secular trends when estimating the effect of the intervention, we repeated the adjusted analyses as a simple pre/post analysis using data only from the intervention ED. Since instituting an early, parallel, multidisciplinary approach to patient assessment usually reserved for the very highest acuity patients might be expected to have the greatest effect in lower acuity patients—i.e. patients whose evaluation clinical teams might otherwise assign a lower priority—we conducted exploratory sensitivity analyses that repeated the difference-in-differences analysis of the primary outcome among subjects with (1) low triage acuity scores (3–5 on the 5-point CTAS scale) and (2) initial qSOFA scores of 0. Finally, we also repeated the primary analysis among (1) patients who had a blood culture collected while in the ED and (2) patients with an explicit discharge diagnosis for severe sepsis or septic shock based on International Classification of Disease Clinical Modification version 9 (995.92 or 785.52) or version 10 (R65.20 or R65.21) discharge diagnosis codes.

Analyses were performed using Stata version 16.0 (StataCorp LLC, College Station, TX). A two-tailed p value ≤0.05 was considered statistically significant. *A priori* power estimates assuming 400 eligible intervention ED patients and 700 eligible control ED patients and a 15-minute decrease in pre-intervention versus post-intervention door-to-antibiotic time at the control EDs estimated 80% power for our difference-in-differences analysis to detect a 26-minute post-intervention change in door-to-antibiotic time at the intervention site.

## Results

**Fig 2:**
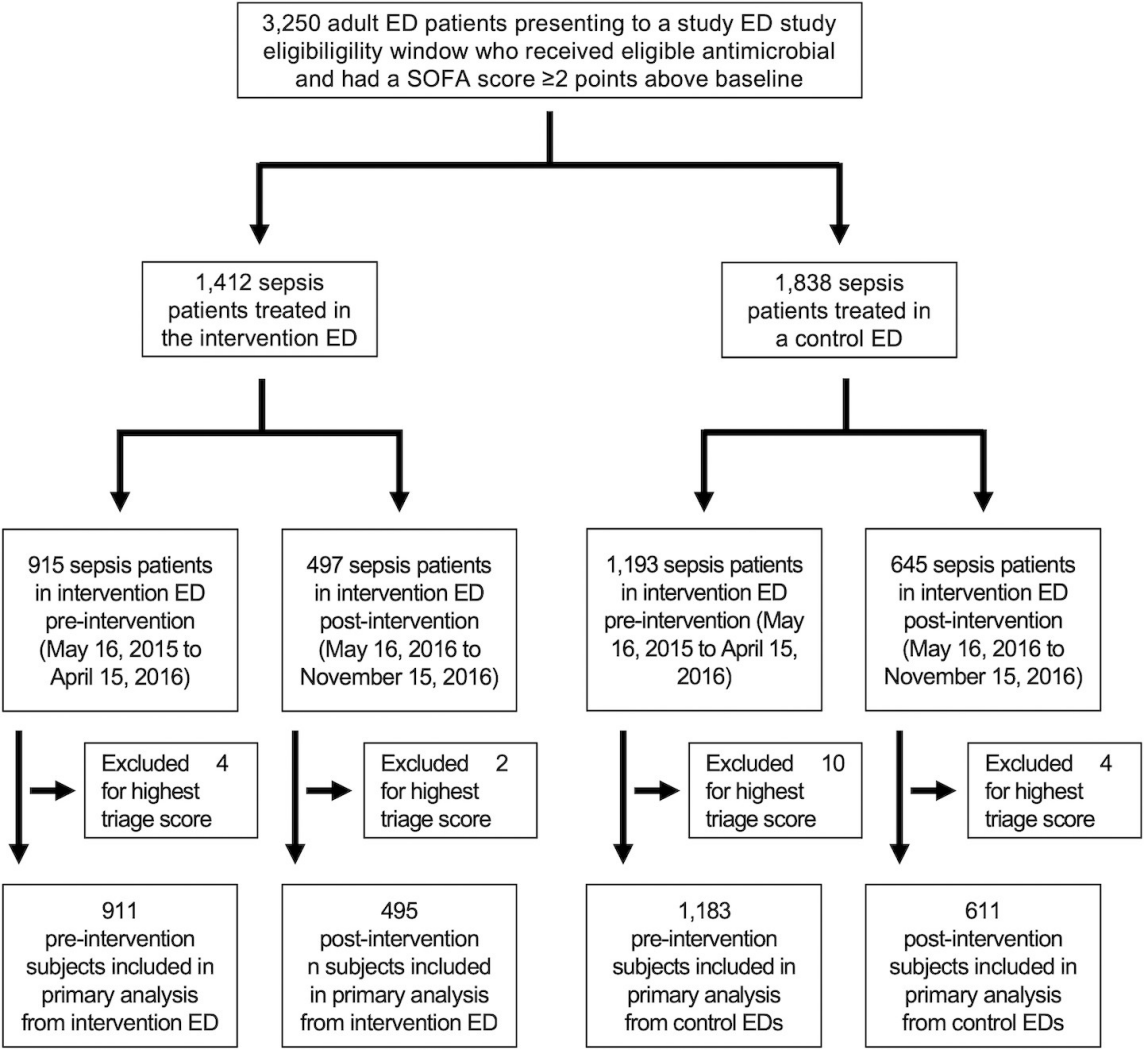
CONSORT-style diagram.

Among 11,094 ED encounters at the intervention hospital from mid-May through late September, 6,277 (57%) had a swarm activated, 6,277 (57%) of all ED patients had a swarm event activated. Median daily adherence to the swarming protocol was 58% (interquartile range 50–66%). A total of 3,250 adult patients presented to a study ED during the pre- or post-implementation period who had a SOFA score ≥2 points above baseline and received an eligible antibiotic in the ED (Fig 2). Twenty patients assigned the highest triage score were excluded from analysis. Data for calculation of baseline SOFA was available for 32% of subjects; a baseline SOFA score of 0 was assigned to other subjects. In the random sample of 100 included subjects, 91% had confirmed infection on final adjudication.

Included subjects presenting to the intervention ED before versus after implementation of the “swarming” intervention were similar, as were subjects presenting in the pre- and post-implementation periods at control EDs (Table 2). Compared to the intervention ED, control ED patients were older, more likely to be female, and less likely to be of Hispanic ethnicity or non-white race.

**Table 2:** Subject demographic and clinical characteristics.

Parameter	Intervention ED							Control EDs							P value for intervention vs. control ED comparison
	Pre-intervention		Post-intervention		P value	Overall		Pre-intervention		Post-intervention		P value	Overall		
													(N = 1,824)		
	(N = 911)		(N = 495)			(N = 1,406)		(N = 1,183)		(N = 641)					
Age	56	(39–70)	56	(39–70)	0.74	56	(39–70)	63	(45–76)	61	(43–76)	0.26	62	(44–76)	<0.001
Female sex	474	(52.0%)	251	(50.7%)	0.64	725	(51.6%)	687	(58.1%)	377	(58.8%)	0.76	1,064	(58.3%)	<0.001
Non-white race or Hispanic ethnicity	197	(21.6%)	117	(23.6%)	0.39	314	(22.3%)	109	(9.2%)	68	(10.6%)	0.34	177	(9.7%)	<0.001
Triage acuity score					0.023							0.018			0.015
Emergent (score 2)	259	(28.4%)	108	(21.8%)		367	(26.1%)	363	(30.7%)	166	(25.9%)		529	(29.0%)	
Urgent (score 3)	618	(67.9%)	364	(73.5%)		982	(69.8%)	785	(66.4%)	464	(72.4%)		1,249	(68.5%)	
Semi- or non-urgent (score 4–5)	34	(3.7%)	23	(4.7%)		57	(4.1%)	35	(2.9%)	11	(1.7%)		57	(2.5%)	
Arrival to ED via ambulance	225	(24.7%)	95	(19.2)	0.019	320	(22.8%)	179	(15.1%)	104	(16.2%)	0.54	283	(15.5%)	<0.001
First-available clinical parameters															
Systolic blood pressure (mmHg)	129	(115–147)	128	(115–144)	0.26	129	(115–146)	133	(117–150)	133	(117–150)	0.78	133	(117–150)	<0.001
Temperature (°C)	37.4	(36.6–38.4)	37.0	(36.3–38.0)	<0.001	37.0	(36.5–37.8)	37.0	(36.5–37.8)	37.1	(36.5–37.9)	0.12	37.0	(36.5–37.8)	0.001
Glasgow Coma Scale score ≤14	55	(6.0%)	24	(4.8%)	0.36	79	(5.6%)	61	(5.2%)	37	(5.8%)	0.58	98	(5.4%)	0.76
White blood cell count (1000/μL)	11.6	(6.4–15.5)	11.5	(8.5–15.4)	0.66	11.5	(8.4–15.5)	11.2	(8.0–15.3)	11.5	(8.5–15.4)	0.19	11.3	(8.2–15.4)	0.41
Lactate checked and ≥2 mmol/dL	231	(25.4%)	138	(27.9%)	0.31	369	(26.2)	316	(26.7%)	164	(25.6%)	0.60	480	(26.3%)	0.96
Charlson Comorbidity Index	2	(0–5)	2	(0–5)	0.02	2	(0–5)	2	(1–5)	2	(1–5)	0.30	2	(1–5)	0.29
SOFA score	3	(2–5)	3	(2–4)	0.33	3	(2–5)	3	(2–4)	3	(2–4)	0.56	3	(2–4)	0.15

Abbreviations: ED, emergency department; SOFA score, Sequential (Sepsis-Associated) Organ Failure Assessment score

Data are shown as median (interquartile range) or N (%).

Median door-to-antibiotic time at the intervention ED was 157 (IQR 107–216) minutes during the pre-implementation baseline period and 151 (IQR 102–205) minutes during the post-implementation period (p = 0.11, Table 3). Inpatient mortality was low both before (2.1%) and after (1.4%) the intervention. Other unadjusted outcomes are shown in Table 3.

**Table 3:** Unadjusted study outcomes by study site and intervention period.

Outcome	Intervention ED				Control EDs			
	Pre-intervention (N = 911)		Post-intervention N = 495)		Pre-intervention (N = 1,183)		Post-intervention (N = 641)	
Door-to-antibiotic time (minutes)	157	(107–216)	151	(102–205)	153	(111–210)	149	(106–207)
Door-to-antibiotic time ≤3 hours	568	(62.3%)	325	(65.7%)	752	(63.6%)	430	(67.1%)
ED length of stay (minutes)	251	(200–322)	246	(194–315)	245	(201–315)	244	(194–308)
Door-to-physician evaluation (minutes)	22	(12–33)	19	(13–30)	17	(10–28)	16	(9–27)
Inpatient mortality	19	(2.9%)	7	(1.4%)	23	(1.9%)	11	(1.7%)

Abbreviations: ED, emergency department

Data are shown as median (interquartile range) or N (%).

In the primary analysis employing multivariable adjustment for patient characteristics within a difference-in-differences framework to account for secular trends in outcomes unrelated to the study intervention, there was no difference in door-to-antibiotic times after care reorganization at the intervention ED (-7 minutes, 95% CI -20 to +6 minutes, p = 0.29). Other secondary outcomes were also unchanged after implementation of swarming (Table 4). Swarming implementation was also not associated with changes in door-to-antibiotic time in sensitivity analysis focused on patients with low triage scores and qSOFA score of 0 (Table 5). Results were also similar when analyses were restricted to patients who had a blood culture obtained in the ED or had an explicit discharge diagnosis of severe sepsis or septic shock.

**Table 4:** Estimated effect of ED “swarming” based on adjusted difference-in-differences analysis and simple multivariable regression.

Outcome	Adjusted difference-in-differences regression^a^			Simple multivariable regression^a^^,^^b^		
	Effect estimate		P value	Effect estimate		P value
	(95% CI)			(95% CI)		
Door-to-antibiotic time (minutes)	-7.1	(-20.4–6.1)	0.29	-13.8	(-24.2 –-3.4)	0.009
Door-to-antibiotic time ≤3 hours (OR)	1.11	(0.81–1.52)	0.51	1.34	(1.05–1.70)	0.019
ED length of stay (minutes)	-9.5	(-25.9–7.0)	0.26	-17.7	(-31.0 –-4.5)	0.009
Door-to-physician evaluation (minutes)	-0.8	(-3.3–1.8)	0.55	-2.8	(-4.4 –-1.1)	0.001
Inpatient mortality (OR)	0.81	(0.25–2.61)	0.72	0.75	(0.2–1.95)	0.55

Abbreviations: CI, confidence interval; ED, emergency department

^a^ Adjusted for age, sex, Charlson Comorbidity Index, initial GCS ≤14, initial systolic blood pressure, ED triage acuity score, and arrival to ED via ambulance.

^b^ Analysis restricted to subjects (N = 1,406) presenting to the intervention ED.

**Table 5:** Sensitivity analyses of ED “swarming’s” estimated effect on door-to-antibiotic time.

Outcome	N	Difference-in-differences estimate of post-intervention change in door to antibiotic time^a^ (minutes, [95% CI])		P value
Triage acuity urgent (3), semi-urgent (4), or non-urgent (5)	2,334	-4.4	(-20.3–11.6)	0.59
Quick SOFA score = 0	2,034	-12.2	(-20.9–4.5)	0.15
Blood culture collected in ED	1,629	-3.4	(-20.4–13.6)	0.69
Explicit sepsis discharge diagnosis code	547	1.9	(-22.6–26.4)	0.88

Abbreviations: CI, confidence interval; ED, emergency department

^a^ Adjusted for age, sex, Charlson Comorbidity Index, initial GCS ≤14, initial systolic blood pressure, ED triage acuity score, and arrival to ED via ambulance

Compared to difference-in-differences analyses, simple pre/post multivariable analyses focused only on the intervention ED yielded larger effect estimates, most of which were statistically significant (Table 4). In particular, door-to-antibiotic time was estimated to decrease by 14 minutes (95% CI 3–24 minutes, p = 0.009).

## Discussion

In this study, we used ED care reorganization for mid- and low-acuity patients as a natural experiment to estimate the stand-alone effect of “swarming” on sepsis care processes and outcomes. We found no significant change in door-to-antibiotic times based on a rigorous quasi-experimental design that used contemporaneous controls to account for changes in the outcome unrelated to the study intervention. Analyses that adjusted only for patient characteristics and used a simple pre/post design, by contrast, would have suggested that the care redesign was associated with significantly shorter door-to-treatment times.

Unlike stroke and myocardial infarction—serious conditions presenting to the ED for which outcomes are similarly dependent on treatment timing—sepsis has a much more subtle clinical presentation and no simple, standardized test to quickly confirm or exclude the diagnosis. Sepsis case identification instead requires clinician judgment synthesizing patients’ symptoms, history and risk factors, physical examination, laboratory test results, and imaging findings. Driven partly by regulatory mandates and public reporting requirements [7,24], many hospitals are currently investing substantial resources in efforts to improve sepsis care delivery.

Swarming has been proposed as a method to improve the efficiency of ED care by reducing duplicative serial evaluations and helping the treating team develop a shared mental model regarding the patient’s evaluation and treatment [14,15]. We wondered whether this relatively simple, low-intensity, and broadly-targeted intervention intended to expedite evaluation for all mid- and low-acuity ED patients would have a moderate but nonetheless meaningful impact on sepsis patients’ door-to-antibiotic time. While we cannot rule out the possibility that alternative low-intensity interventions or 100% adherence to swarming would have shown benefit, our findings suggest that simply bringing the clinical team together at the bedside may be insufficient to improve ED-based care delivery for patients with sepsis. Instead, more intensive, focused, and multimodal interventions may be required to meaningfully improve sepsis care. Besides broadening membership of the swarming team (in particular, by adding a pharmacist), a more effective sepsis-specific protocol might integrate automated tools for very early identification of patients at risk of sepsis, standardized protocols to facilitate rapid completion of diagnostic tests, ongoing evaluation of barriers to sepsis protocol adoption and effectiveness, and/or individual case review and clinician feedback. However, stand-alone team-based interventions like swarming could also have beneficial effects on other outcomes—including patients’ care experience, clinical team members’ satisfaction, and medical errors— which we were unable to measure in the present analysis. While prior data suggest care processes targeted by swarming are important determinants of antibiotic timing [10,11,25–27], the absence of statistically significant association could reflect swarming’s failure to influence other rate-limiting processing preceding antibiotic initiation (e.g. antibiotic order review and fulfilment by pharmacy).

Strengths of this study include an analytic approach more conducive to causal inference than simple pre/post analysis and an approach to confounder adjustment consistent with recent guidelines [28,29]. In fact, use of simple pre/post design for this analysis would have suggested the swarming intervention was associated with a moderate but statistically significant decrease in door-to-antibiotic time. This discrepancy between the results of simple pre/post analysis and our quasi-experimental analysis highlights the methodological problems with studies of system-level interventions that fail to account for secular trends.

Our study has several limitations. The studied EDs shared a common sepsis protocol throughout the study, but unrecognized interventions affecting sepsis care that were unique to the control EDs could have led to under-estimation of swarming’s effect at the intervention hospital. Other potential issues derive from our guideline-adherent approach to sepsis case identification. About 1 in 10 study subjects did not prove to have infection on final review, a value similar to the true-positive infection rate (88%) reported by Rhee *et al*. in a study where the overall positive-predictive value of electronic sepsis case identification was 70% [2]. However, analyzing patients with suspected infection at the time of ED evaluation is consistent with sepsis consensus definitions and, more importantly given the questions investigated here, the clinical decisions confronting ED clinicians. To wit, the final determination of sepsis status is usually unavailable at the time ED clinicians must make therapeutic decisions. Our method for identifying patients for study enrollment may also have misclassified some patients with simple infection due to either (1) a lack of causal association with concurrent acute organ failure or (2) overestimation of acute organ failure severity resulting from missing data for calculation of baseline SOFA scores. Finally, we excluded sepsis patients whose infection was unrecognized or untreated during the ED stays, as well as patients whose organ failure was not detectable or not present before ED discharge. We were unable to assess whether swarming altered the likelihood of sepsis diagnosis, infection treatment, or organ failure detection during the ED encounter.

The most important limitation of this study, however, was our inability to identify whether specific ED patients actually received swarm-based care. Uptake was fairly high during the initial months after implementation but anecdotally may have waned toward the end of 2016. For this reason, we *a priori* closed the data analysis period in mid-November, 2016. While our study should accurately reflect the impact associated with a pragmatic implementation of ED swarming, we cannot exclude the possibility that results would have differed if the analysis had been restricted to patients who received a swarm-based evaluation or if additional resources had been dedicated to promoting and maintaining adherence to the swarming model. Appropriately designed and controlled prospective hybrid implementation/effectiveness trials could not only quantify *whether* interventions like swarming improve sepsis care but would also provide empiric data on *why* such interventions succeed or fail.

## Conclusion

Introduction of “swarm”-based evaluation for mid- and lower-acuity ED patients was not associated with improvements in door-to-antibiotic time for patients with sepsis after accounting for secular trends in sepsis care processes and outcomes.
